# Silver Flowerlike Structures for Surface-Enhanced Raman Spectroscopy

**DOI:** 10.3390/nano11123184

**Published:** 2021-11-24

**Authors:** Gitchka G. Tsutsumanova, Neno D. Todorov, Stoyan C. Russev, Miroslav V. Abrashev, Victor G. Ivanov, Alexey V. Lukoyanov

**Affiliations:** 1 Faculty of Physics, Sofia University, 5 James Bourchier Blvd., 1164 Sofia, Bulgaria; ggt@phys.uni-sofia.bg (G.G.T.); neno@phys.uni-sofia.bg (N.D.T.); scr@phys.uni-sofia.bg (S.C.R.); mvabr@phys.uni-sofia.bg (M.V.A.); 2 M.N. Miheev Institute of Metal Physics, Ural Branch, Russian Academy of Sciences, S. Kovalevskaya St. 18, 620108 Ekaterinburg, Russia; lukoyanov@imp.uran.ru; 3 Ural Federal University, Mira St. 19, 620002 Ekaterinburg, Russia

**Keywords:** SERS, SERS substrates, nanoflowers, surface plasmon, nanocavity resonator

## Abstract

Micro- and nanoflowers are a class of materials composed of particles with high surface-to-volume ratio. They have been extensively studied in the last decade due to simple preparation protocols and promising applications in biosensing, as drug delivery agents, for water purification, and so on. Flowerlike objects, due to their highly irregular surface, may act also as plasmonic materials, providing resonant coupling between optical waves and surface plasmon excitations. This fact allows us to infer the possibility to use micro- and nanoflowers as effective surface-enhanced Raman scattering (SERS) substrate materials. Here, we report on the design and Raman enhancement properties of silver flowerlike structures, deposited on aluminum surface. A simple and cost-effective fabrication method is described, which leads to SERS substrates of high developed surface area. The morphology of the silver flowers on a nanoscale is characterized by self-organized quasiperiodic stacks of nanosheets, which act as plasmonic cavity resonators. The substrates were tested against rhodamine-6G (R6G) water solutions of concentration varying between 10^−3^ M and 10^−7^ M. Optimal SERS enhancement factors of up to 10^5^ were established at R6G concentrations in the 10^−6^–10^−7^ M range.

## 1. Introduction

Surface-enhanced Raman scattering (SERS) is a powerful analytical technique, which undergoes substantial evolution over the years [1,2,3,4]. Currently, there is a broad range of SERS applications, among which single molecule detection [5,6,7], biosensing [8,9,10], gas detection (“artificial nose”) [11], etc. There are two principal mechanisms that lead to SERS effect—chemical and electromagnetic (EM) enhancement. The former is a result of adsorbate-substrate bonding interactions resulting in surface electronic states of high Raman polarizability [12]. The EM enhancement stems from resonant coupling between the incident or scattered photons with the surface plasmon (SP) or localized plasmon (LP) excitations on a non-planar metallic surface [13]. Typically, noble metals (silver, gold) are the best candidates for SERS substrate materials since they provide SP/LP resonance frequencies in the visible range in combination with low electromagnetic losses [14].

As a rule, the EM enhancement is not uniform over the substrate, but is localized on specific ‘hot spots’, where local electric field created by SP/LP excitations overwhelms by orders of magnitude the amplitude of the incident light wave. Hot spots are usually formed due to the lightning-rod effect—e.g., in the gap between closely separated nanoparticles [7,15,16], or near sharp metallic tips [17,18] (a SERS clone known as tip-enhanced Raman spectroscopy—TERS). Since the morphology of the substrate plays a key role in the EM enhancement, much effort is being paid to the SERS substrate design. Currently, many preparation techniques are being used, like electrochemically roughened surfaces [19,20], deposition of metallic nanoparticles on a dielectric support/substrate [21,22,23,24,25,26], micrometer sized metallic sheets [27,28], nanoholes [29], nanorods and nanowires [30], etc. Special attention is being paid to periodic nanoscale arrays, since they allow for finer tuning of the SP resonance in a desirable spectral range [31,32,33,34].

Yet another important factor in SERS is the effective area of the active surface. Generally, elevated Raman signal could be retrieved from substrates of larger developed area due to the higher number of adsorbed molecules within irradiated surface. In this aspect micro- and nanoflowers (NF) are an interesting new class of materials [35,36]. Typically, the NF is an object composed of several individual sheets (leafs) organized in specific pattern, oftentimes resembling a real flower. Due to the large surface-to-volume ratio, NFs find promising applications in biosensors, catalysis, drug delivery, etc. [36]. The highly irregular surface is favorable for effective light-SP/LP coupling and hot spots formation, which infers for NF applications as plasmonic materials and SERS substrates. The SERS properties of different nanoflower structures has been explored in a number of works within the last decade [37,38,39,40]. Liang et al. [37] have reported the synthesis of silver flowerlike nanoparticles of sizes 500 nm–2 μm with chemically roughened surface. This morphology allows for formation of a large number of hot spots, and SERS EF of 10^7^–10^8^ has been established for single NFs, while an order of magnitude higher enhancement has been found in NF aggregates. Another approach is based upon composites, consisting of a flowerlike ‘skeleton’ with high effective area, covered with noble-metal nanoparticles, which are responsible for the hot spot formation. For example, hexagonal arrays of flower-shaped Au@Ag structures have been investigated by Xing et al. [38]. The Au@Ag NFs are based on aggregates of intersecting silver nanosheets, which are covered with gold nanoparticles. SERS EF up to 10^7^ and a detection limit down to 10^–11^ M for thiram solution have been reported for that system. More recently, similar SERS performance has been established in ZnO/Ag nanoflower composites, which consist of a flowerlike ZnO base uniformly covered with Ag nanoparticles [40]. Typically, for the nanoparticle-based SERS substrates, the Raman signal enhancement has been found localized around isolated hot spots. Yet another type of flower-based SERS substrates has been reported for flowerlike aggregates of micrometer-size silver crystals [39]. This type of substrates has been proven effective for detection of 4-aminothiophenol in trace concentrations, although the EF has not been quantified. The SERS effect has been qualitatively ascribed to electrodynamic enhancement in the vicinity of the sharp microcrystal edges and in the gaps between them.

The principal aim of the present work was to further investigate the capabilities of metallic flowerlike structures as SERS substrate materials. Our intent was to combine the large surface-to-volume ratio of the NFs with the evenly distributed SERS response characteristic of the artificial periodic nanostructures. Objects of investigation were the earlier reported silver flowers (‘roses’) deposited on aluminum surface [41], due to the simple chemical route of preparation and the possibility of ordering in 2D arrays. In particular, we were interested on the influence of the nanoscale morphology of the silver roses on their SERS enhancement. As will be shown below, the as prepared silver flowers display self-organized assemblies of silver nanosheets (leaves) with subwavelength periodicity, which infers for an effective SP interaction with the exciting/scattered optical field.

We describe the preparation and characterization of the substrates, as well as the details of SERS measurements in Section 2. The experimental results are reported and discussed in Section 3. The summary and conclusions of our research are formulated in Section 4. Appendix A describes the details of calculation of the dispersion of the propagating SP waves in a periodic assembly of silver nanosheets.

## 2. Materials and Methods

### 2.1. Preparation and Characterization of the SERS Substrates

Silver nitrate, ammonia (25%), and glucose monohydrate were supplied from Merck (Kenilworth, NJ, USA). Aluminum foil (99%, 100 μm thickness) was obtained from Honeywell Riedel-de Haën (Charlotte, NC, USA) and R6G (R4127, dye content 95%)—from Sigma Aldrich (provided by Merck Bulgaria EAD, Sofia, Bulgaria).

Silver flowerlike structures were grown on aluminum foil via standard silver mirror reaction. The procedure follows that, described in [42]: 2.5 mL ammonia (1.1 mol/L) was added to 10 mL silver nitrate aqueous solution (0.12 mol/L) until the AgOH/Ag_2_O precipitate just disappeared. The Ag(NH_3_)_2_OH solution was then mixed with 15 mL aqueous solution of glucose (0.56 mol/L). The samples were positioned on the bottom of a petri dish with the as prepared solution. After 60 min they were removed from the solution, rinsed with distilled water and dried in air. During the growth process of the silver flowers, some of the substrates, referred to hereafter as ‘n’ series, were kept at room temperature. The other samples, referred to as ‘s’ series, were put on a hot plate and kept at constant temperature of 40 °C.

**Figure 1:**
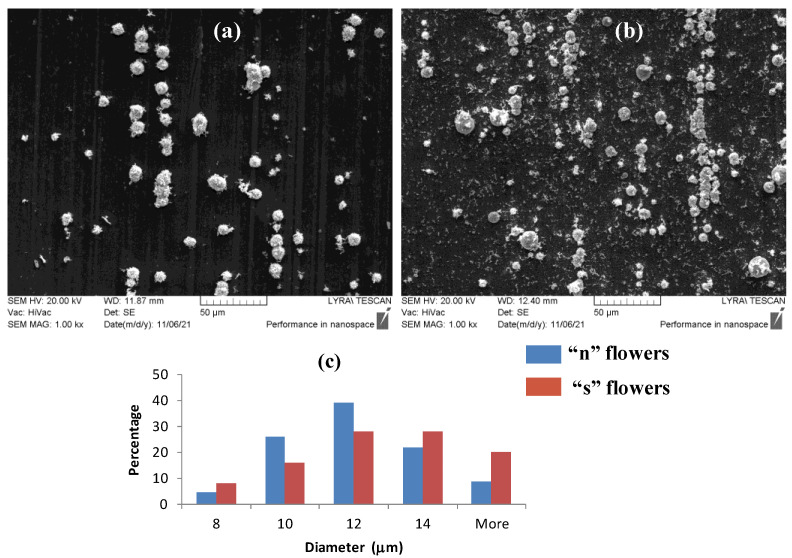
Scanning electron micrographs of the silver flowers on ‘n’ substrates (**a**), and ‘s’ substrates (**b**) at 1000× magnification. (**c**) Histograms with 2-μm bin range of diameter distribution of the silver flowers on both types of substrates.

The morphology of the as prepared samples was inspected with a scanning electron microscope (LYRA I XMU, Tescan (Brno-Kohoutovice, Czech Republic)) equipped with an EDX microanalyzer (Quantax, Bruker). Representative large scale SEM micrographs of the ‘n’ and ‘s’ substrates are shown in Figure 1a,b, respectively. The silver flowers grow along areas of clean aluminum surface, visible as brighter grooves, where the thin oxidized aluminum layer is mechanically disrupted. A controllable growth of the same type of structures is also possible on a clean aluminum surface pre-sealed with a thin PMMA film. The growth centers are created by selective etching of PMMA layer by means of electron-beam lithography, as described in [41]. Figure 1c,d, represent the diameter distribution of the silver flowers for both types of substrates. The average flower diameter is approximately the same for both types of substrates: (11.2 ± 2.0) μm for the ‘n’-series and (12.0 ± 2.5) μm for the ‘s’-series, respectively.

A number of lone nanosized silver sheets are dispersed on the aluminum around the silver flowers on the ‘s’-substrates due to the convection in the solution during the growth on the hot plate. For the ‘n’-substrates, almost no isolated silver sheets are found on the substrate.

**Figure 2:**
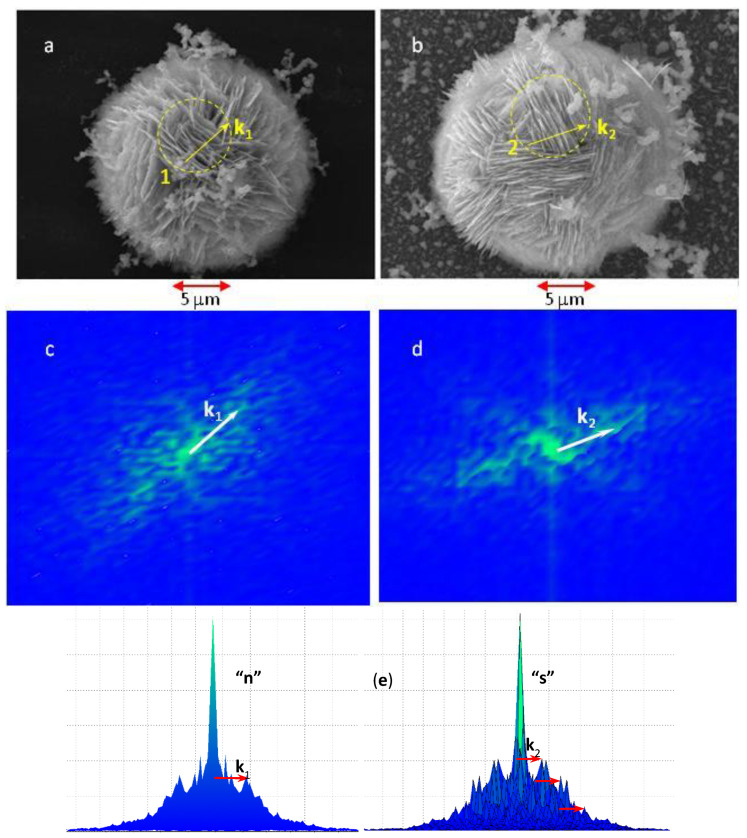
Scanning electron micrographs of the silver flowers on ‘n’ substrates (**a**), and ‘s’ substrates (**b**). FT amplitude patterns of areas 1 (**c**) and 2 (**d**). Vectors **k**_1_ and **k**_2_ are the corresponding propagation wavevectors of structures in areas 1 and 2. (**e**) A plot of the FT amplitude along *Y*-direction for the two areas. Overtones of the wavevector **k**_2_ are also marked.

Figure 2a,b represent typical SEM images of individual silver flowers on the ‘n’ and ‘s’ substrates, respectively. Flowers consist of silver flakes, most of which are organized in quasiperiodic arrays of 10–15 almost parallel vertically standing sheets, as exemplified by the marked areas 1 and 2 in the figure. The Fourier transform (FT) amplitude patterns of the selected areas of the microscopic images display diffuse but resolvable peaks corresponding to the propagation wavevector **k** of each array, as indicated in Figure 2c,d. The corresponding spatial period Λ of a given stack is, therefore

Λ *=* 2π/|**k**|. (1)

The as estimated period varies among different arrays of a same silver flower, as well as among different flowers of a same substrate. Thus, we calculated Λ on groups of 3–4 arrays on each of the investigated flowers, and then performed paired two-tail Student’s statistical tests between samples of values corresponding to different flowers of the same substrate. We established that the propagation periods, found on different flowers on the same type of substrate, are likely to originate from the same statistical distribution with a statistical significance (*p*-value) in the 18–30% range. The corresponding estimated mean values and standard deviations are (580 ± 60) nm for the ‘n’ substrates and (390 ± 30) nm for the ‘s’ substrates. These values corroborate the visual perception of denser packing of silver sheets on the ‘s’ flowers, compared to the ‘n’-flowers, as seen in Figure 2a,b. Arrays on the ‘s’-flowers display better spatial coherence than the arrays formed on the ‘n’-flowers, as evidenced by Figure 2e, where the peaks corresponding to the second and the third overtone of the propagating vector **k** are clearly resolvable. The discrete FT of the images was performed by means of a specially written MATLAB script, which implements the following FT convention: $\mathrm{FT}\left[y\right]\left(k\right)=\underset{n}{\sum }y_{n}\exp\left(-ik\cdot x_{n}\right)$, where the sum runs over all pixels of the investigated portion of the SEM image, and $y_{n}\;$ represents the image brightness at the *n*-th pixel. Prior to FT analysis the SEM image brightness was normalized to a 0–1 scale, where 0 corresponds to the minimum recorded brightness and 1—to the brightness maximum. In that case the silver sheet edges correspond to y≈1, while the voids give a negligible contribution. Accordingly, the filling factor *F* of the arrays, i.e., the volume fraction of the silver sheets, has been estimated as: *F* = *A*_0_/*N*, where *A*_0_ is the amplitude of the sharp central maximum (**k** = 0, see Figure 2e) of the FT pattern and *N* is the number of pixels in the investigated portion of the SEM image. Approximately the same value of *F =* 0.15 ± 0.02 was found for both types of silver flowers. Thus, the average thickness *b = F*Λ of the silver sheets was estimated to 87 nm for the ‘n’-flowers and 59 nm for the ‘s’-flowers, respectively.

### 2.2. Methodology of the SERS Measurements

Rhodamine-6G solution of 10^–3^ M concentration was prepared by dilution of 50.3 mg of R6G in 100 mL of deionized (DI) water. Solutions of progressively lower concentrations, ranging from 10^−4^ M to 10^−7^ M, were obtained by four consecutive steps of dilution of the starting 10^−3^ M solution. Where suitable, we will also refer to the solutions by their dilution order (DO), equal to the number of dilution steps. Thus, the molar concentration *C* of the solution is related to DO by the equation

*C =* 10^−(3+DO)^. (2)

Two substrate samples, one of each series—‘s’ and ‘n’, were immersed for 1 h in solutions of all available R6G concentrations. After that the substrates were rinsed with DI water and dried in dark atmosphere at room temperature for 12 h. The Raman measurements on the as treated substrates were performed on a micro-Raman spectrometer LabRAM HR800 (Horiba/Jobin Yvon, Edison, NJ, USA) at room temperature with a He-Ne laser (632.8 nm) as an excitation source. A × 50 objective was used both to focus the incident laser beam on a spot of diameter around 5 μm, and to collect the backward scattered light. Since pure R6G is a weak Raman scatterer, reference spectra from R6G solutions were recorded from a liquid cell placed in a retroreflector compartment, which additionally enhances the Raman signal. For a given concentration, Raman spectra were collected from two areas on each type of substrate—on the silver flowers and on the aluminum surface between them. According to the SEM images (see Figure 1) the focused laser spot on the silver flowers illuminates the lateral edges of 10–15 silver sheets. For each substrate, treated in R6G, spectra were collected from at least three randomly chosen silver flowers and three random spots on the bare aluminum surface.

In order to quantify the SERS properties of the substrates, we calculated the normalized intensity *I*(ν), associated with the ν-th R6G vibrational mode, as

*I*(ν) = *S*(ν)/(*CP*_L_), (3)

where *S*(ν) is the integrated signal obtained by a Lorentzian fit of the corresponding band in the Raman spectrum of the bulk solution or of the substrate treated with that solution, *P*_L_ is the excitation laser power, and *C* is the R6G concentration in the solution. The analytic SERS enhancement factor for a specific Raman mode ν was defined according to [43]

EF(ν) = *I*_SERS_(ν)/*I*_sol_(ν), (4)

where *I*_sol_(ν) and *I*_SERS_(ν) are the normalized mode intensities recorded in the bulk of a referent solution and from the silver flower, respectively.

## 3. Results and Discussion

**Figure 3:**
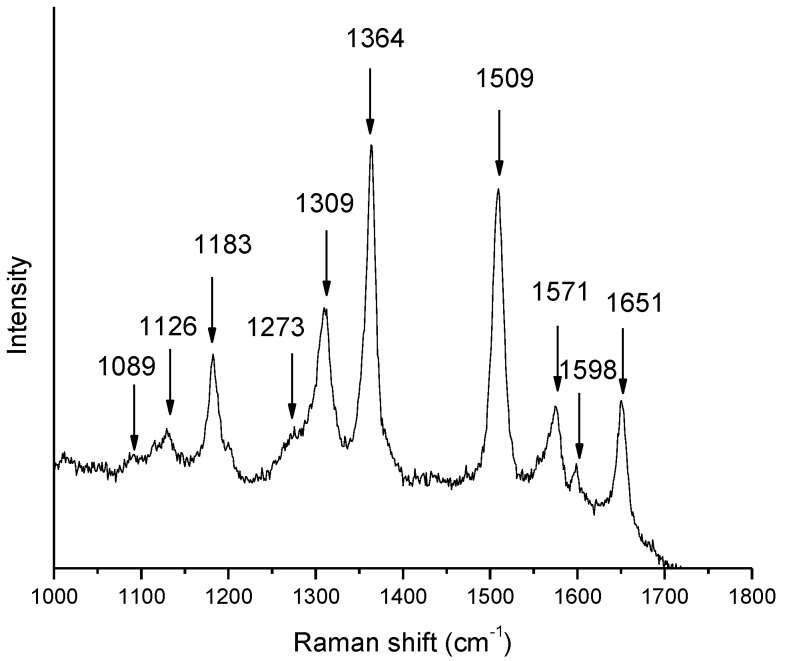
Raman spectrum of 10^−3^ M R6G solution.

**Figure 4:**
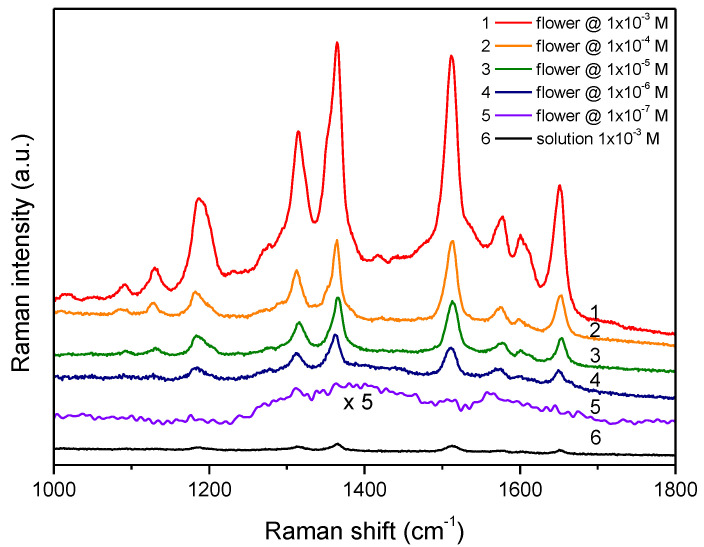
Spectra from silver roses, treated in R6G solutions of different concentration (1–5). Spectrum #6 is taken from the bulk of the 10^−3^ M R6G solution. Spectra correspond to the raw Raman signal and are not normalized to the solution concentration.

Figure 3 shows a representative Raman spectrum taken from the bulk of the starting 10^−3^ M solution. The most intense vibrational modes of R6G are easily identifiable and their frequencies are marked on the figure. The SERS properties of the substrates are clearly manifested in Figure 4, where the spectrum of the bulk 10^−3^ M solution (#6) is compared to the spectra 1–5 from silver flowers on ‘n’ substrates treated in R6G solutions of progressively decreasing concentration. The spectra are represented on the same intensity scale and are shifted vertically for clarity. Obviously, the detected Raman signal in spectra 1–5 decreases monotonously with the decrease of the concentration of R6G solution. All R6G vibrational modes are clearly resolved in spectra 1–4, except in spectrum 5, corresponding to smallest R6G concentration, where the spectral lines are masked to a large extent by the spectral noise. Qualitatively similar results have been established for the substrates of the ‘s’ series.

SERS EF for different R6G vibrational modes, calculated according to Equations (3) and (4) for all concentrations, are listed in Table 1. In order to obtain stable fits, the intensity of the R6G spectral lines for samples, treated in the most diluted 10^−7^ M solution, were estimated by deconvolution of Lorentzian profiles with spectral positions and widths fixed to the corresponding values obtained for the 10^–6^ M solution. The last row of the table lists the corresponding spectrum-average enhancement factor (SAEF) defined as $\mathrm{SAEF}=\underset{\nu }{\sum }I_{\mathrm{SERS}}\left(\nu \right)/\underset{\nu }{\sum }I_{\mathrm{sol}}\left(\nu \right)$, where the sum runs over all detected vibrational modes. Each entry in the table is a statistical average calculated on a sample of at least three randomly chosen silver flowers.

**Figure 5:**
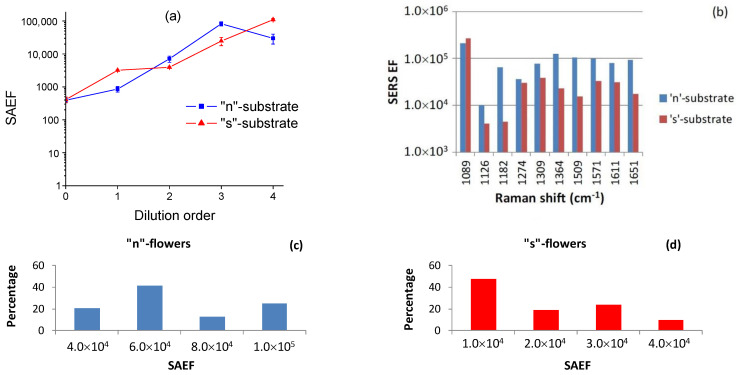
(**a**) Spectrum-averaged enhancement factor (SAEF) for ‘n’- and ‘s’-substrates treated with R6G solutions of different concentrations. (**b**) SERS enhancement factors for different R6G modes on silver flowers treated in a 10^−6^ M solution. Histograms of SAEF recorded on different silver flowers treated in a 10^–6^ M solution: ‘n’-flowers (**c**) and ‘s’-flowers (**d**).

Figure 5a represents the concentration dependence of SAEF. For both types of samples the SERS enhancement systematically increases at low concentrations, reaching optimum values of ≈10^5^ for samples treated in 10^−6^–10^−7^ M R6G solutions. Figure 5b shows the spectral distribution of the EF over different R6G modes. Again, the two types of samples give qualitatively similar results, showing a maximum at low wavenumbers, a pronounced dip for the mode at 1126 cm^−1^ followed by gradual increase for the modes around ≈ 1300 and 1600 cm^−1^.

The concentration dependence of SERS EF is a phenomenon found for different types of substrates and a wide range of analytes [44,45]. Typically, SERS is less effective for higher concentration due to the saturation of adsorption of the analyte molecules on the substrate. Respectively, optimal EF is obtained at smaller concentrations, when monomolecular layer is formed on the substrate [44]. In that case the most favorable conditions are met for both the chemical and the electrodynamic enhancement mechanisms, since adsorbed molecules are in a direct chemical contact with the substrate, and at the same time sense the highest local electric field.

Figure 5c,d shows the statistical distribution of SAEF recorded on an ensemble of silver flowers on each type of substrates, treated in 10^−6^ M solution. The data demonstrate a very good reproducibility of the SERS enhancement among different silver flowers. The spread of SAEF values for a given substrate is less than an order of magnitude, and is related to a large extent to the variations of the focusing conditions during measurements. We did not establish any statistically significant correlation between the flower size or aggregation and the SERS signal. This fact suggests that the SERS’ effect on silver flowers is related mainly to their submicron morphology, as will be discussed in details below.

**Figure 6:**
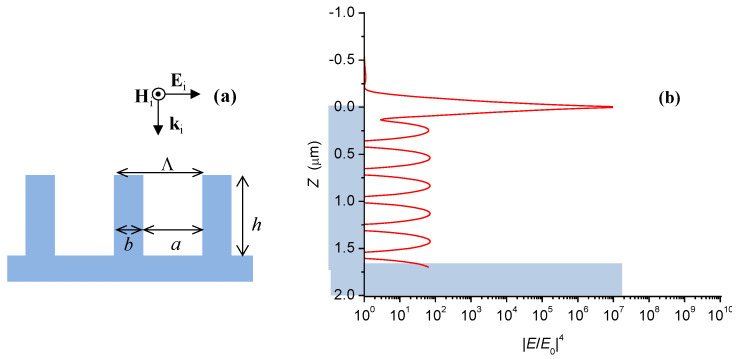
(**a**) Schematic model of a rectangular metallic grid. A TM polarized wave is incident normally on the grating, along the wavevector **k***_i_*. The electric field **E***_i_* of the wave is perpendicular to the metallic sheets (i.e., along the propagation direction of the grid); the magnetic field **H***_i_* is parallel to the edges of the grooves. (**b**) Local electrodynamic enhancement factor $g\left(r\right)={\left|E/E_{0}\right|}^{4}$ over vertical surface of the silver sheet under resonant conditions. *Z*-coordinate represents the depth below the edge of the silver sheet.

As already shown in Figure 2, the two types of silver flowers consist of self-organized quasiperiodic arrays, which could be modeled as rectangular gratings of vertical sheets of a thickness *b*, separated by grooves (voids) of a width *a =* Λ − *b*, where Λ is the grating period, as illustrated in Figure 6a. The depth *h* of the grooves is of the order of 2–3 μm, i.e., it exceeds several times the grating period. Therefore, this structure is equivalent to a system of coupled nanocavity resonators, which transmits the SP waves along the inner silver-air interface in depth of the grooves. The resonant frequencies of the cavity are approximately given by the standing wave condition for an open-end resonator

(5) $$2h=\left(m+\phi /\left(2\pi \right)\right)\lambda_{\mathrm{SP}},$$

where $\lambda_{\mathrm{SP}}$ is the frequency-dependent SP wavelength, and ϕ is the phaseshift of the wave reflected from the bottom of the grooves, and *m* is the number of wave nodes [46,47]. The SP in a metallic grating is excited by an electromagnetic wave of TM polarization, i.e., with a magnetic field parallel to the groove edges, and an electric field having component parallel to the grating propagation vector (see Figure 6a). As seen in Figure 2, any single silver flower is composed of several arrays whose propagation vectors span different directions. Therefore, irrespectively of the polarization of the laser excitation, there are arrays coupled to the electric field of the incident wave.

Theoretical models of SP excitations in single nanocavities [46,47] and in periodic rectangular gratings [48,49] suggest that the strongest electrodynamic enhancement takes place near the parallel edges of the metallic sheets, which could act as effective SERS hot spots [50]. In contrast to hot-spots found in nanoparticle-based SERS substrates—which are essentially point-like (0D) objects—the hot-spots in metallic gratings have linear (1D) character, and can accommodate a larger number of adsorbed molecules. The availability of a large number of groove edges illuminated by the laser excitation implies that the EM enhancement in the silver flowers proceeds mainly through the formation of linear hot-spots. However, for SP resonances transmitted in depth of the grooves, electric field enhancement takes places also over the inner metal-dielectric interface, near the crests of the standing SP wave. Numerical simulations evidence that molecules adsorbed deeply in the grating grooves also contribute to the overall SERS signal [50].

In order to theoretically assess the relevance of the suggested resonator-cavity model for the silver flowers, we performed Fourier modal method (FMM) [51] calculations for the rectangular grid shown in Figure 6a. The details of the calculations are described in Appendix A. The local EM enhancement of the Raman signal was estimated as $g\left(r\right)={\left|E\left(r\right)/E_{0}\right|}^{4}$ where *E*(**r**) is the amplitude of the local electric field at point **r** of the metal–air interface and *E*_0_ is the amplitude of the incident wave [50]. Figure 6b shows the calculated distribution of g(r) along the vertical surfaces of the silver sheets, corresponding to the parameters of the “s”-flowers and for the wavelength λ_0_ = 633 nm of the incident wave. The height *h* = 1.7 μm of the silver sheets is chosen to match the resonance condition (4) for *m =* 5 and ϕ=26.3° at the silver-air interface. In full agreement with [50], we established the formation of a hot-spot near the groove edge, as evident from the sharp maximum of $g_{\max}\approx 9.4\times 10^{6}$ at *Z =* 0. Deeper along the silver sheet, there is a sequence of equidistant maxima of $g\approx 1\times 10^{2}$, which correspond to the crests of the standing SP wave. Although the maximum signal enhancement is close to 10^7^ near the hot spot, the experimentally observed EFs are by 2 orders of magnitude lower because the R6G molecules are adsorbed over the whole surface if the silver leaves. The averaged electrodynamic enhancement over the entire silver–air boundary, including the upper ridge of the silver sheet was calculated at $\langle g\left(r\right)\rangle \approx 8.7\times 10^{4}$, which is very close to the experimentally established SERS EFs at low R6G concentration. We established that a hot spot with similar electrodynamic enhancement is formed even at off-resonant conditions due to the lightning-rod effect near the sharp edge of the silver sheet. Thus, the overall SERS EF is practically independent on the variation of the grooves’ depth *h*. Similar calculations for the geometric parameters of the ‘n’-flowers revealed slightly smaller values of $g_{\max}\approx 7.2\times 10^{6}$ and $\langle g\left(r\right)\rangle \approx 6.8\times 10^{4}$. We have to stress that our model does not account for three important factors, which can also significantly affect the experimentally measured EFs:

1. Uneven distribution of the adsorbed molecules over the surface of the silver sheets. Plausibly, the R6G molecules are more likely to settle at the upper parts of the sheets, i.e., near the edges where the local electrodynamic enhancement is higher. This fact may explain qualitatively the experimental EF values superior to 10^5^, which are higher than the calculated averaged EFs.
2. Local field enhancement at the contact area between neighboring nanosheet arrays of different orientation. In these areas, the contacting nanosheets make an angle close to 60°, which is also favorable for formation of linear hot spots.
3. Field enhancement around defects on the surface of the silver sheets.

## 4. Summary and Conclusions

In summary, we report the preparation and characterization of SERS substrates based on silver flowerlike particles of high surface-to-volume ratio. The nanoscale morphology of the silver flowers is dominated by self-assembled arrays of vertically standing metallic sheets of subwavelength periodicity. The recorded SERS enhancement factors on the silver flowers show excellent reproducibility and reach the highest values of ∝10^5^ at lowest analyte concentrations of 10^−6^–10^−7^ M.

The theoretical simulations reveal that the major contribution to the SERS enhancement stems from the groove edges, which act as effective ‘hot lines’. This effect is stronger for the substrates of the ‘s’-series, where groove edges are closer compared to the edges on the ‘n’-substartes. The periodic nanosheet arrays, however, form coupled resonator cavities, which guide the surface plasmon waves in depth of the silver flowers, providing additional electric filed enhancement over entire area of the silver nanosheets.

The work opens prospects for further research, like optimization of the preparation conditions for obtaining predefined morphology of the nanosheet arrays within silver flowers, testing the substrates against different analytes, and investigation of the possibility for multiple usage/washability of the substrates. In particular, it would be of interest to elaborate fabrication conditions for silver flowers with smaller-period nanosheet arrays, which provide higher concentration of linear hot spots. The good reproducibility of the SERS signal in combination with elaborated technology of “seeding” of silver flowers in predefined positions—including in ordered patterns [41]—infers for promising applications of this type or SERS substrates in biosensing and in medical analysis.

## Figures and Tables

**Table 1:** The SERS EF for the silver flowers for different R6G modes as a function of the dilution order of the R6G solution. The corresponding spectrum-averaged enhancement factors (SAEF) are shown on the last row. The left and the right columns for each concentration correspond to the ‘n’ and ‘s’ flowers, respectively.

Mode Frequency (cm^–1^)	Dilution Order									
	0 EF (×10^2^)		1 EF (×10^3^)		2 EF (×10^3^)		3 EF (×10^4^)		4 EF (×10^5^)	
1089	1.6	7.4	1.1	34	5.9	26	21	27	1.3	8.2
1126	0.6	1.5	0.3	0.4	1.5	0.4	1.0	0.4	0.07	0.6
1183	6.4	6.3	1.1	5.3	9.7	2.2	6.4	0.5	0.07	0.8
1273	5.9	5.8	0.2	2.8	1.3	2.5	3.6	3.0	0.29	2.7
1309	3.2	3.3	0.9	4.6	5.9	3.6	7.7	3.9	0.25	0.9
1364	5.5	4.7	1.2	5.7	10	6.4	13	2.3	0.50	1.6
1509	5.9	5.2	1.5	3.6	12	4.8	11	1.6	0.29	0.3
1571	4.2	3.6	0.6	2.5	7.1	2.9	9.9	3.3	0.32	1.7
1598	1.8	1.2	0.3	2.2	2.5	2.1	8.1	3.1	0.32	0.7
1651	4.3	4.3	1.3	3.2	7.8	4.0	9.4	1.8	0.26	0.8
SAEF	4.0 × 10^2^	4.1 × 10^2^	8.6 × 10^2^	3.2 × 10^3^	7.1 × 10^3^	4.0 × 10^3^	8.3 × 10^4^	2.5 × 10^4^	3.0 × 10^4^	1.1 × 10^5^

**Figure A1:**
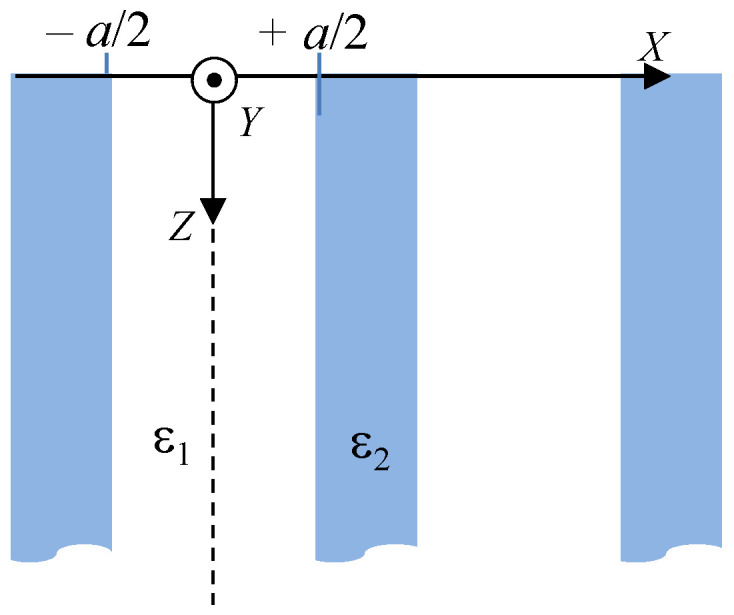
Schematic representation of the periodic MIM structure used in FMM calculations.

**Figure A2:**
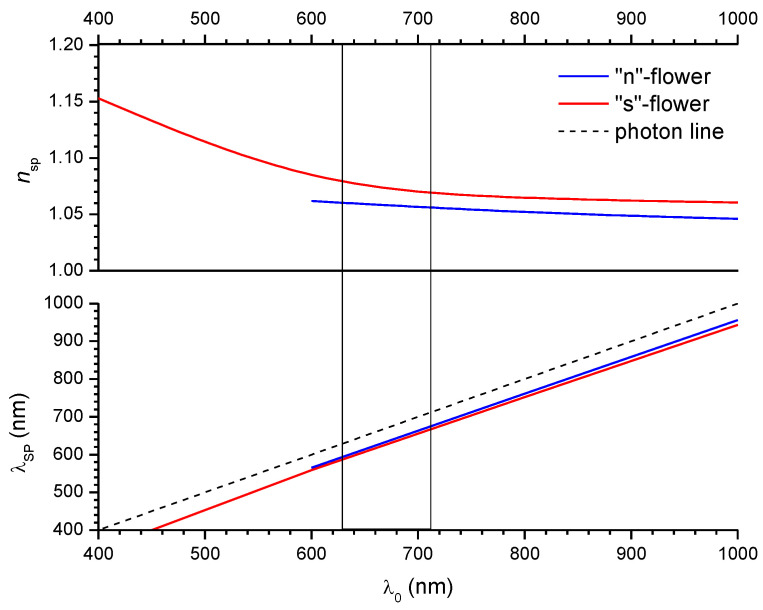
Lower panel: Dependence of the SP wavelength λ_SP_ (lower panel) and the effective SP refraction index *n*_SP_ on the free-space wavelength λ_0_. Only values $\lambda_{0}>\Lambda$ are presented. The Raman spectrum window is marked with vertical lines.

## Data Availability

Publicly available datasets from the Refractive Index Database were analyzed in this study. This data can be found here: https://refractiveindex.info. The source is cited in the References section.

## Appendix A

FMM is among the major calculation schemes for modelling of the electrodynamic response of structures with periodically modulated dielectric permittivity [51]. The method is based on a plane-wave representation of the electric and magnetic field outside the modulated structure. The electromagnetic field inside the modulated region is expanded as a superposition of guided eigenmodes corresponding to an infinite periodic waveguide. The expansion coefficients are found by imposing the field boundary conditions at the interface between the modulated structure and the surrounding medium.

By following FMM the rectangular grating will be modeled as a periodic metal–insulator–metal (MIM) array of infinite waveguides, which transmit SP waves of a wavevector *q* along the *Z*-direction, as shown in Figure A1. The permittivity of the dielectric medium is ε_1_ and the permittivity of the metal is ε_2_. We will neglect the energy losses in the metal and in the dielectric, assuming that ε_d_ and ε_2_ are real.

The SP is excited by a TM polarized plane electromagnetic wave of angular frequency ω and wavelength in free space λ_0_ = 2πc/ω, incident normally onto the grating, i.e., in *Z*-direction (see Figure 6a). The electric field **E***_i_* and the magnetic field **H***_i_* the incident wave are parallel to the X- and *Y*-directions, respectively. Since the X-component of the wavevector of the incident wave is zero, the excited SP waves could be expanded in harmonic components with propagation vector along *X*, multiple of 2π/Λ. Therefore, the electric and magnetic field of the SP wave satisfy the periodic boundary conditions E(x)=E(x+Λ) and E(x)=E(x+Λ), respectively. We assume that ω < ω_p_ (the plasma frequency of the metal), which means that ε_2_ < 0 and the electromagnetic field falls off exponentially inside the metallic slabs.

Since in our case $\Lambda <\lambda_{0}$ we will follow the approach in [47] for calculating the dispersion relation in a single MIM cavity of a subwavelength width *a* and a much bigger depth *h*. Due to the periodic boundary conditions, we consider filed behavior only in the ‘elementary cell’ x∈[−a/2;Λ−a/2], which includes the [−a/2;+a/2] dielectric cavity and the neighboring metallic sheet to the right. The non-zero components of the magnetic field **H** and the electric field **E** in the dielectric (labeled with index 1), and in the metal (labeled with index 2), which satisfy the Maxwell equations, and are consistent with the polarization of the incident wave, have the form

(A1) $$H_{1y}=F_{1}\cosh\left(\eta_{1}x\right)e^{i\left(qz-\omega t\right)};\;\;H_{2y}=F_{2}\cosh\left(\eta_{2}\left(x-\Lambda /2\right)\right)e^{i\left(qz-\omega t\right)};$$

(A2) $$E_{1z}=\frac{i\eta_{1}F_{1}}{\omega \varepsilon_{1}\varepsilon_{0}}\sinh\left(\eta_{1}x\right)e^{i\left(qz-\omega t\right)};\;\;E_{2z}=\frac{i\eta_{2}F_{2}}{\omega \varepsilon_{2}\varepsilon_{0}}\sinh\left(\eta_{2}\left(x-\Lambda /2\right)\right)e^{i\left(qz-\omega t\right)}.$$

(A3) $$E_{1x}=\frac{qF_{1}}{\omega \varepsilon_{1}\varepsilon_{0}}\cosh\left(\eta_{1}x\right)e^{i\left(qz-\omega t\right)};\;\;E_{2x}=\frac{qF_{2}}{\omega \varepsilon_{2}\varepsilon_{0}}\cosh\left(\eta_{2}\left(x-\Lambda /2\right)\right)e^{i\left(qz-\omega t\right)}.$$

Here, *F*_1_ and *F*_2_ are the complex amplitudes of the magnetic field in the dielectric and in the metal, respectively, $\eta_{1}=\sqrt{q^{2}-{\left(\omega /c\right)}^{2}\varepsilon_{1}}$ and $\eta_{2}=\sqrt{q^{2}-{\left(\omega /c\right)}^{2}\varepsilon_{2}}$ are the corresponding exponential-decay constants. After applying the field boundary conditions:

(A4) $$H_{1y}=H_{2y};\;E_{1z}=E_{2z};\;\varepsilon_{1}E_{1x}=\varepsilon_{2}E_{2x}$$

at the metal–dielectric interface at x=+a/2, we derive the dispersion relation for the SP modes

(A5) $$\frac{\eta_{1}}{\varepsilon_{1}}\tanh\left(\eta_{1}a/2\right)=-\frac{\eta_{2}}{\varepsilon_{2}}\tanh\left(\eta_{2}b/2\right)$$

Equation (A5) reproduces the result for a single MIM cavity [47] in the limit b→∞ at finite a. On the other hand, if both a,b→∞, the classical SP dispersion relation: $q^{2}={\left(\omega /c\right)}^{2}\varepsilon_{1}\varepsilon_{2}/\left(\varepsilon_{1}+\varepsilon_{2}\right)$ for a single MI interface is obtained.

In what follows, we take $\varepsilon_{1}=1$ for the air, while $\varepsilon_{2}$ is the frequency-dependent electric permittivity of silver at ω < ω_p_, taken from [52] and available in numeric form from the Refractive Index Database [53]. The geometric parameters of the model grids, representing the two types of silver flowers, are summarized in Table A1. The lower panel of Figure A2 shows the calculated dependence of the SP wavelength $\lambda_{\mathrm{SP}}=2\pi /q$ on the wavelength $\lambda_{0}=2\pi c/\omega$ of the electromagnetic radiation in the free space at $\lambda_{0}>\Lambda$. The upper panel of the figure presents the wavelength dependence of the effective SP refraction index $n_{\mathrm{SP}}=\lambda_{0}/\lambda_{\mathrm{SP}}$.

**Table A1:** Model grid parameters for the two types of silver flowers. The last row represents the surface-plasmon effective refraction index *n*_SP_, calculated at the wavelength λ_0_ = 663 nm of the incident laser wave.

Parameter	“n”-Flowers	“s”-Flowers
Λ (nm)	580	390
Filing factor *F*	0.15	0.15
*a =* (1 − *F*)Λ (nm)	493	332
*b = F*Λ (nm)	87	58
*n*_SP_ (633 nm)	1.060	1.079

The as-obtained resonator modes were tailored with the plane-wave expansion of the field in the *z* < 0 region (i.e., in air) and in a thick silver base at the bottom (*z > h*).
